# Supplementary figures and images for: Polypeptides derived from α-Synuclein binding partners to prevent α-Synuclein fibrils interaction with and take-up by cells
Source: PLoS One. 2020 Aug 13;15(8):e0237328. doi: 10.1371/journal.pone.0237328 (PMC7425896; doi:10.1371/journal.pone.0237328)

**A**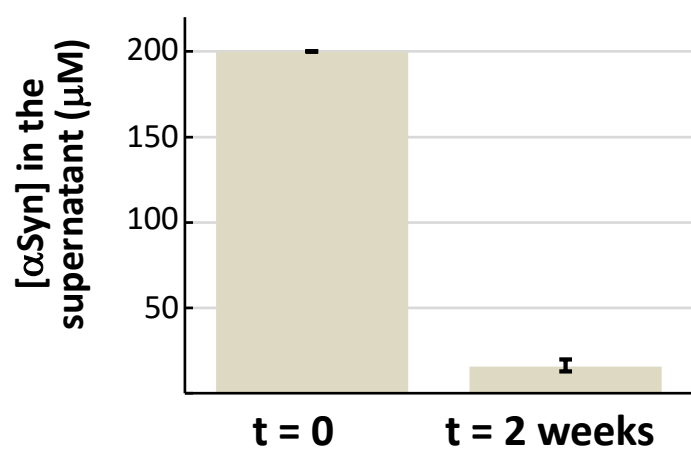**B**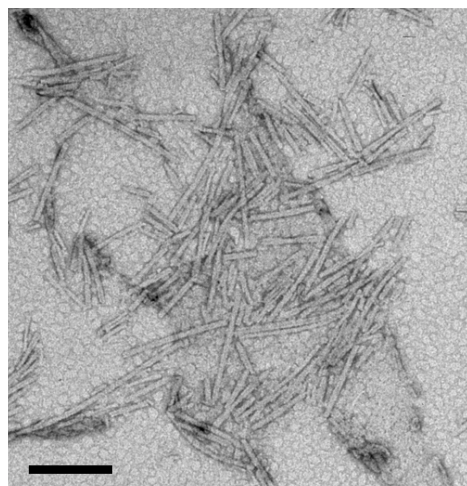**Figure S1**

Supplement: S1 Fig — Monomeric αSyn was assembled for 2 weeks at 200 μM (equivalent monomer concentration), labelled with Alexa488 and sonicated for 1 min, as described in Material & Methods. A, The completion of the aggregation reaction was assessed by measuring the concentration of αSyn in the supernatant at t = 0 and t = 2 weeks. The mean and associated standard deviation values were calculated from 5 independent experiments. B. The fibrillar nature of the resulting aggregates assessed by transmission electron microscopy after negative staining. Scale bar, 200 nm. (PDF) [file pone.0237328.s001.pdf]

**A**

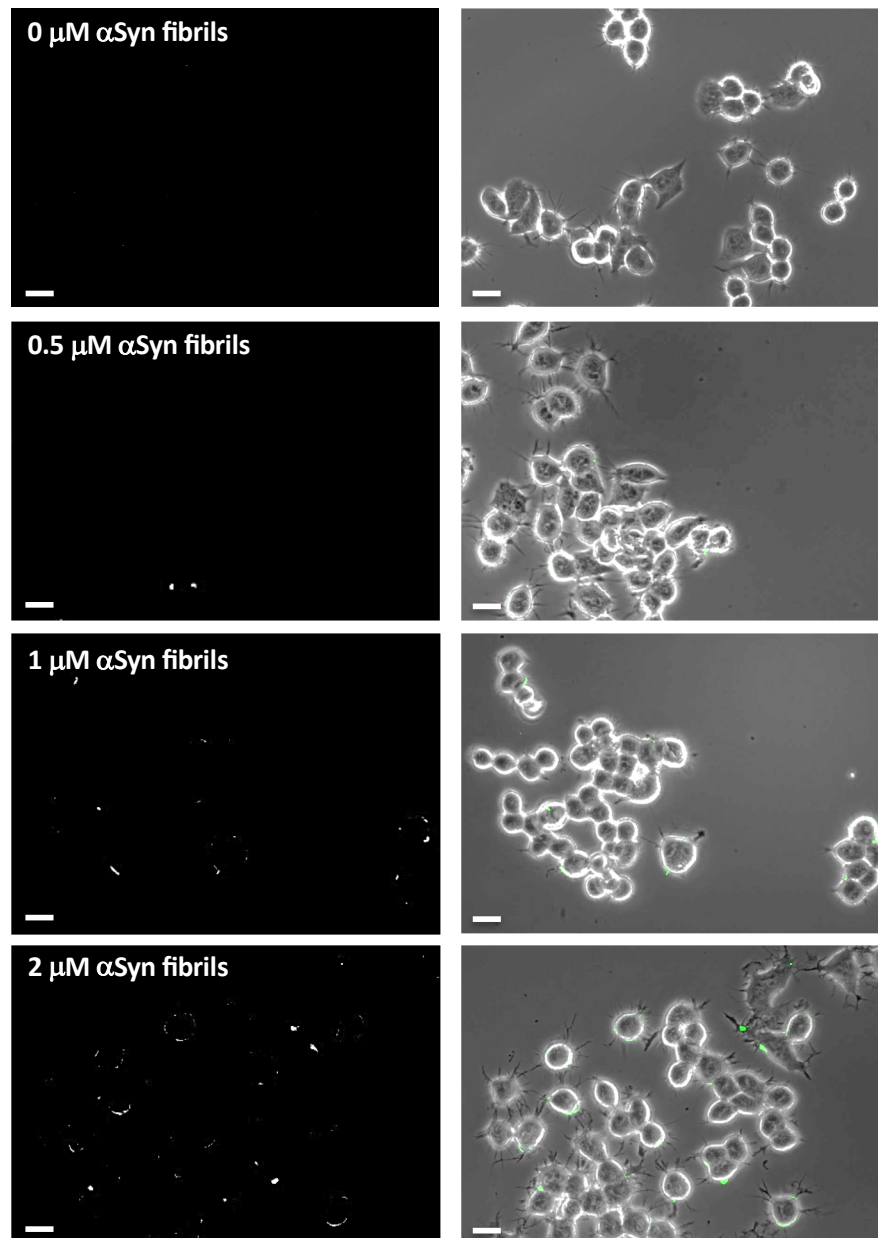

**Figure S2**

**B**

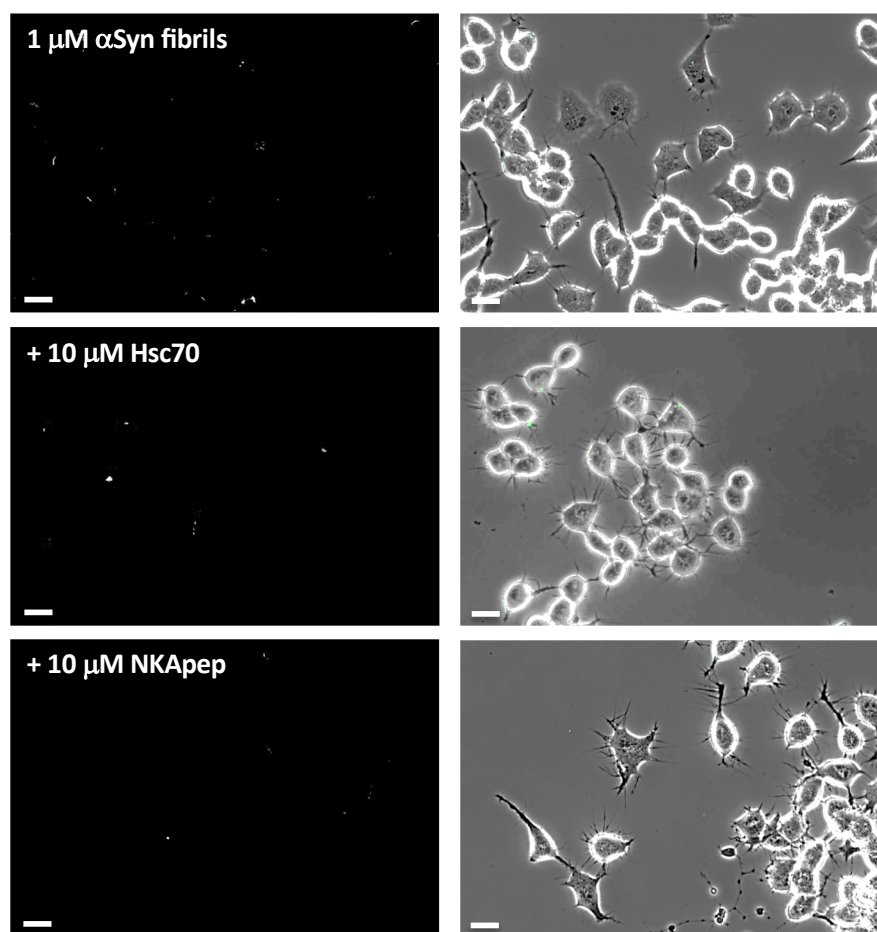

**Figure S2 (continued)**

Supplement: S2 Fig — A, Dose-dependent binding of αSyn fibrils to the plasma membrane of Neuro-2a cells. Neuro-2a cells were imaged after exposure for 30 min to αSyn-Alexa488 fibrils (0–2 μM equivalent monomer concentration) and extensive washing. B, αSyn fibrils binding to the plasma membrane of Neuro-2a cells in the presence or the absence of Hsp70 and NKApep. αSyn-Alexa488 fibrils (1 μM equivalent monomer concentration) were incubated in the absence (top pannels) or in the presence of Hsc70 (10 μM; middle panels) or NKApep (10 μM; bottom panels) in DMEM for 30 min at 37°C. Neuro-2a cells were imaged after exposure to the mixture for 30 min and extensive washing. Scale bars, 20 μM. (PDF) [file pone.0237328.s002.pdf]

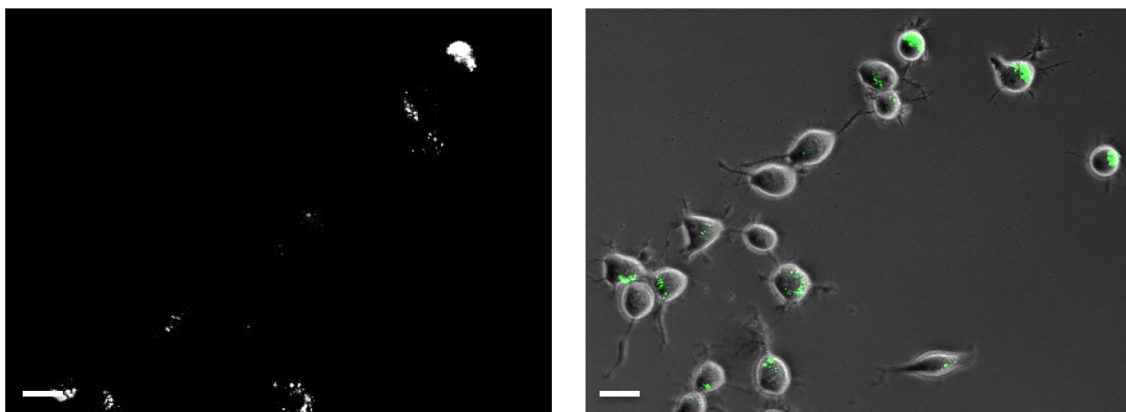

**Figure S3**

Supplement: S3 Fig — Neuro-2a cells were exposed for 4 hours to αSyn-Alexa488 fibrils (1 μM equivalent monomer concentration). The cells were washed twice with serum-free, phenol red-free DMEM then 0.1% Trypan Blue was added to quench the fluorescence of plasma membrane-bound Alexa488-labeled αSyn fibrils. Scale bars, 20 μM. (PDF) [file pone.0237328.s003.pdf]

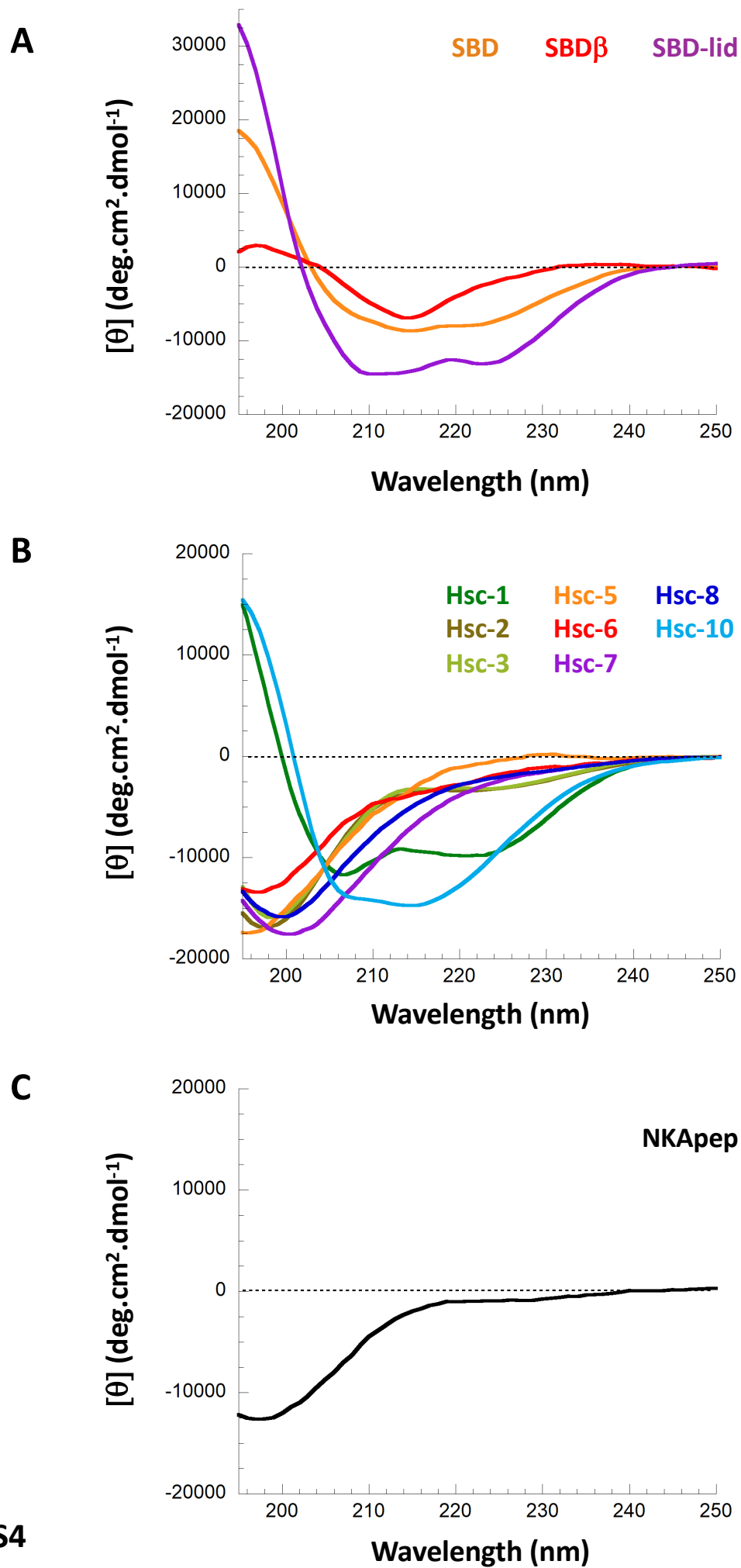

Figure S4

Supplement: S4 Fig — A, Hsc70 domains SBD, SBDβ and SBD-lid. B, Hsc70 peptides. C, NKApep. (PDF) [file pone.0237328.s004.pdf]

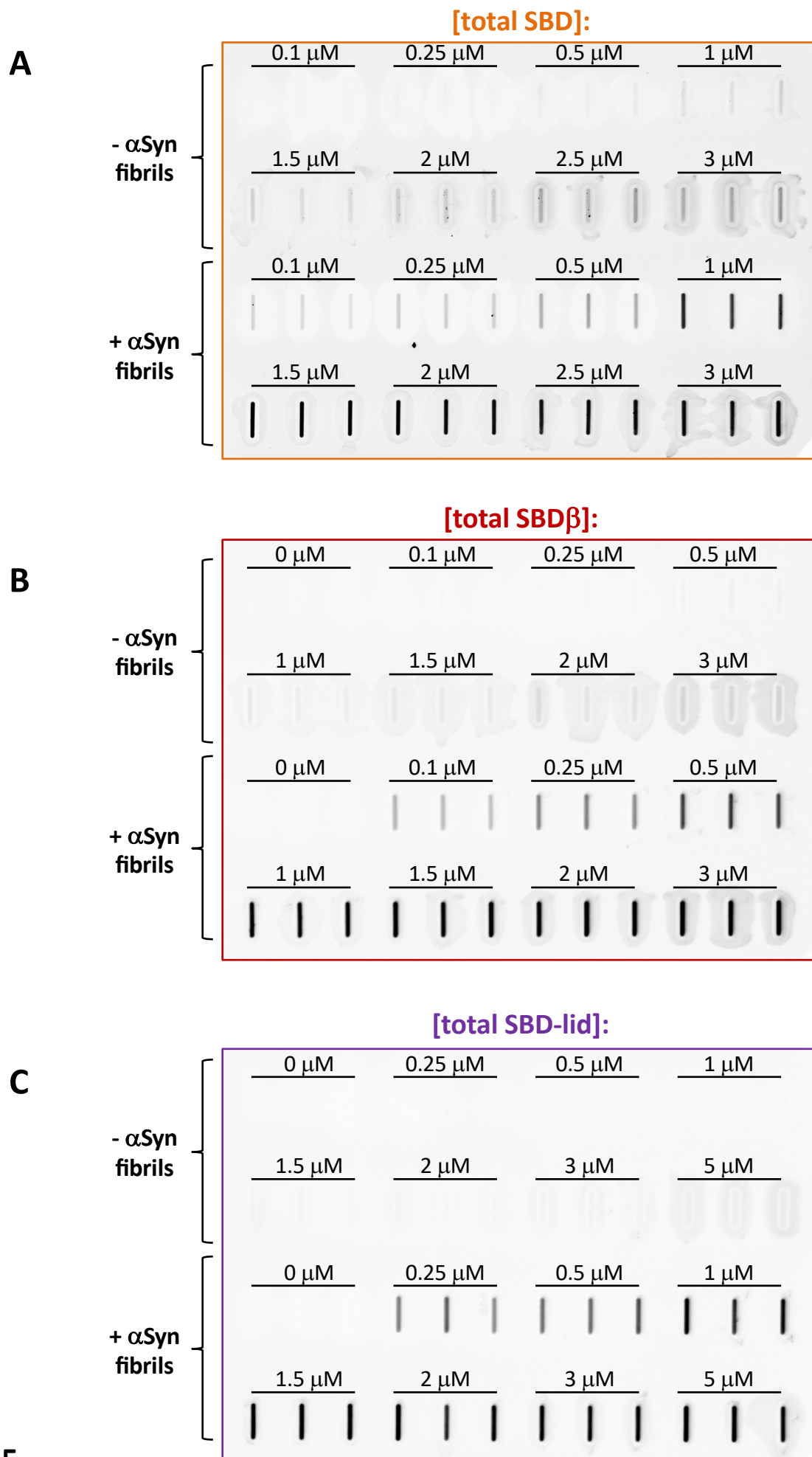

Figure S5

Supplement: S5 Fig — Quantification of SBD-ATTO488 (A), SBDβ-ATTO488 (B) and SBD-lid-ATTO488 (C) binding to αSyn fibrils. ATTO488-labelled Hsc70 SBD domain and sub-domains were diluted with the corresponding unlabelled proteins (at a molar ratio 1:50) to different final concentrations (0–5 μM) and incubated with or without αSyn fibrils (1 μM) for 1h at RT. Each sample was then filtered in triplicate through a cellulose acetate membrane and the amount of ATTO488-labelled Hsc70 domain trapped onto the membrane was quantified. In each case a representative experiment is shown. KD values presented in Fig 4B were derived from 2 to 3 independent experiments. (PDF) [file pone.0237328.s005.pdf]

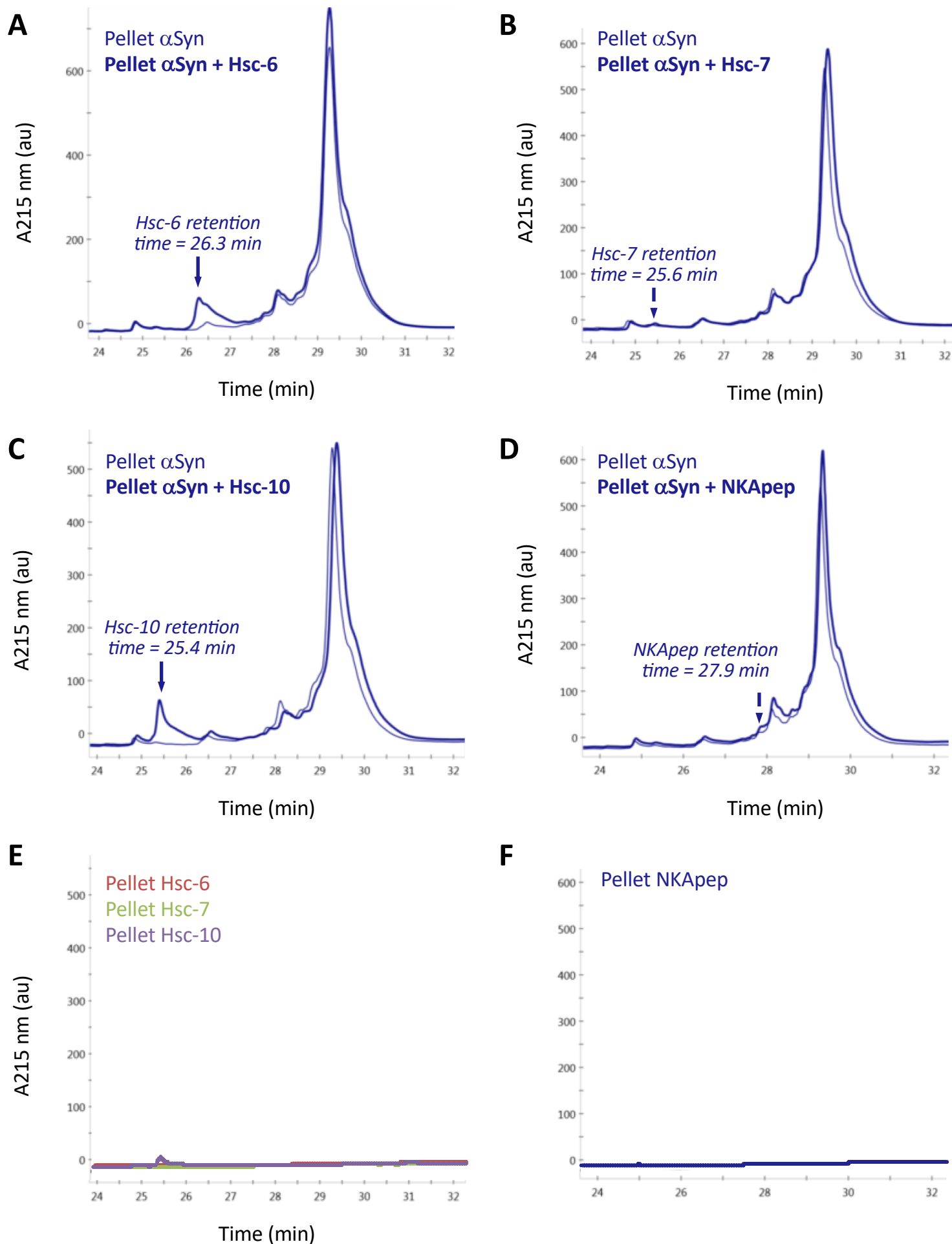

Figure S6

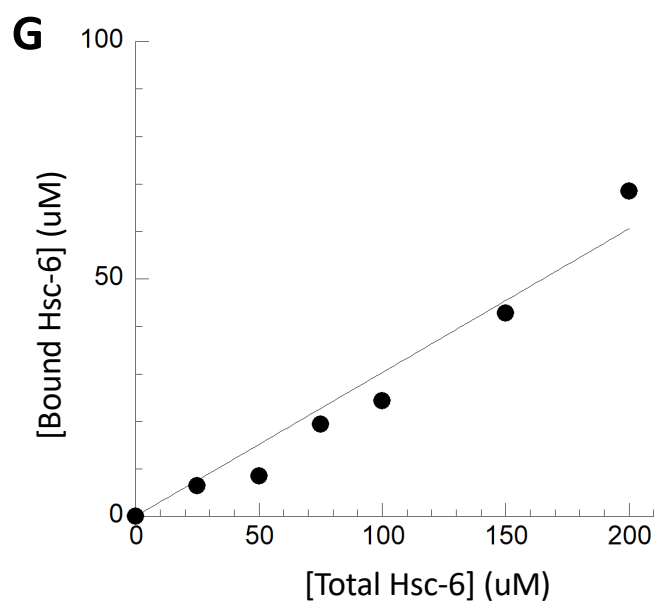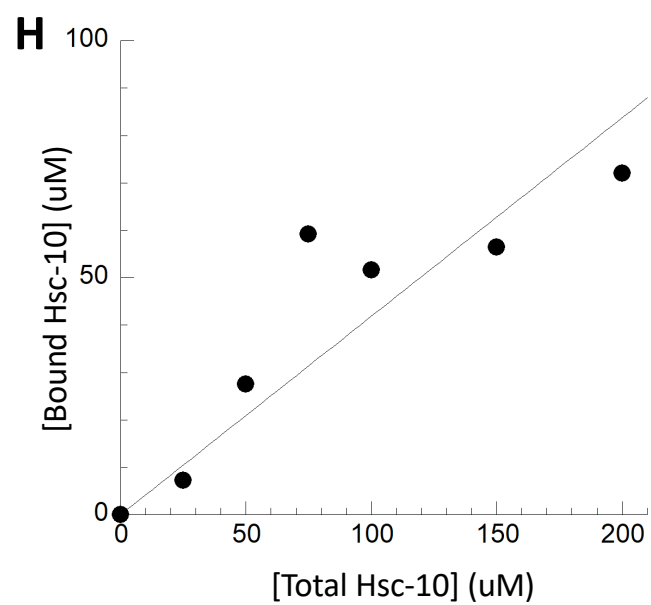

**Figure S6 (continued)**

Supplement: S6 Fig — A-F, αSyn fibrils alone (100 μM), αSyn fibrils (100 μM) and peptides (200 μM) (panels A-D) and Peptides alone (100 μM; panels E and F), were incubated 1h at RT. The samples were centrifuged for 30 min at 20.000g and 20°C. The pellets were dissolved in TFA 100%. After evaporation, the samples were resuspended in TFA 0.1%, and analysed by reversed phase chromatography on a C18 column. The retention time of each peptide was determined by a separate injection of 1 nmole of the peptide and is indicated by an arrow; for Hsc-7 (B) and NKApep (D), the arrow indicating the putative position of the peptide is in dotted line since no peptide was found to be associated with the αSyn pellet. Hsc-6 and Hsc-10 co-sediment with αSyn fibrils, whereas Hsc-7 and NKApep do not. G, H, Determination of Hsc-6 and Hsc-10 - αSyn fibrils KD. Measurements as described above were performed for increasing peptide concentrations (0–200 μM). The amount of αSyn fibrils-bound Hsc-6 and Hsc-10 is plotted against the total peptide concentration. The lines through the data points represent the best fits to a linear function and are drawn for visual guidance only. (PDF) [file pone.0237328.s006.pdf]
