# Supplementary material for: Polypeptides derived from α-Synuclein binding partners to prevent α-Synuclein fibrils interaction with and take-up by cells
Source: PLoS One. 2020 Aug 13;15(8):e0237328. doi: 10.1371/journal.pone.0237328 (PMC7425896; doi:10.1371/journal.pone.0237328)

Gels for Figure 1A

“Hsc70 binds to αSyn fibrils *in vitro*”

SDS-PAGE analysis of the pellet (P) and supernatant (S) fractions of Hsc70 (10 μM), fibrillar αSyn (100 μM), and fibrillar αSyn (100 μM) incubated with Hsc70 (10 μM) for 1 h at RT. 12 μL of a 1:3 dilution in Laemmli were loaded.

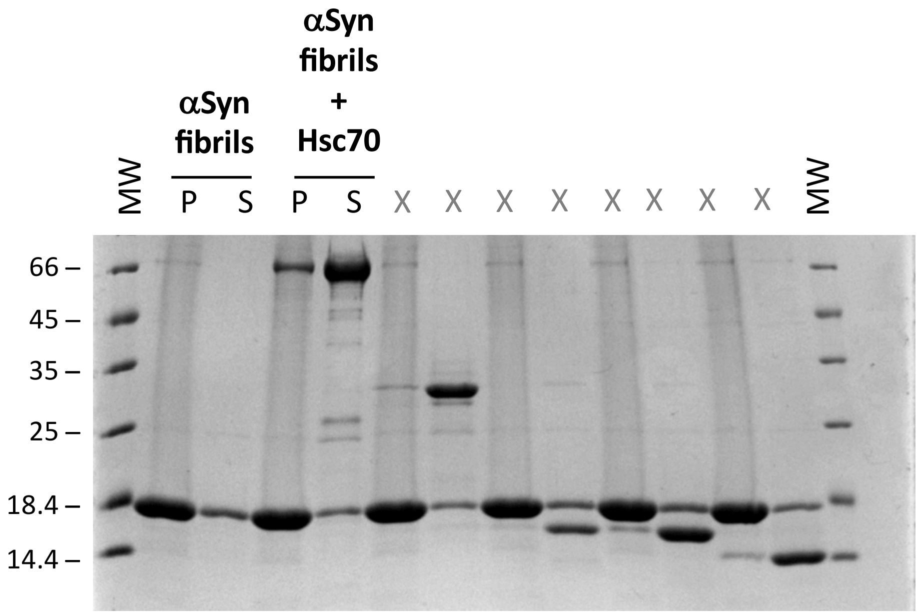

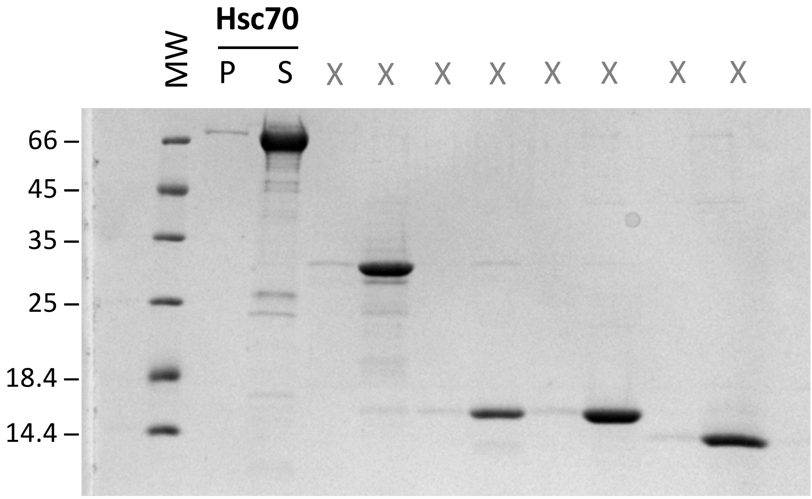

Filter-trap membranes for Figure 1B:

"Quantification of Hsc70-ATTO488 binding to αSyn fibrils using the cellulose acetate filter trap assay"

Hsc70-ATTO488 was diluted with unlabelled Hsc70 (labelled:unlabelled molar ratio of 1:50) to different final concentrations (0-2 μM) and incubated with or without αSyn fibrils (1 μM) for 1h at RT. Each sample was filtered in triplicate through a cellulose acetate membrane and the amount of Hsc70-ATTO488 trapped onto the membrane was quantified.

Three independent experiments were performed, and the corresponding three filter-trap membranes are shown. The filter-trap membrane shown in Figure 1B corresponds to replicate n°3.

Replicate n°1

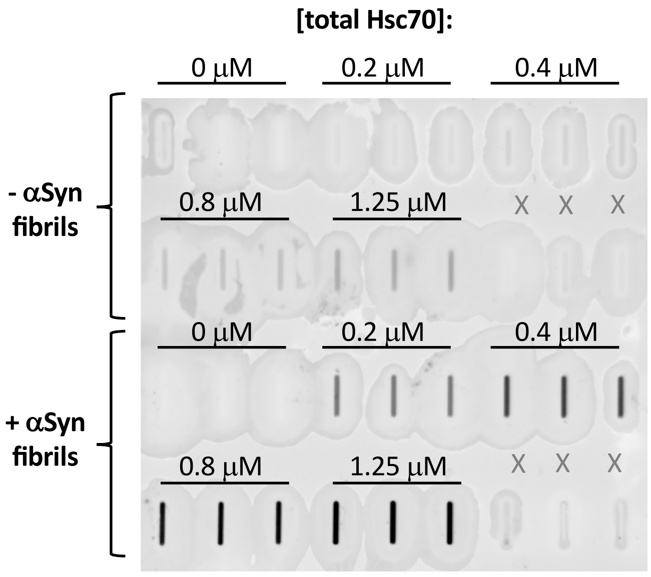

Replicate n°2

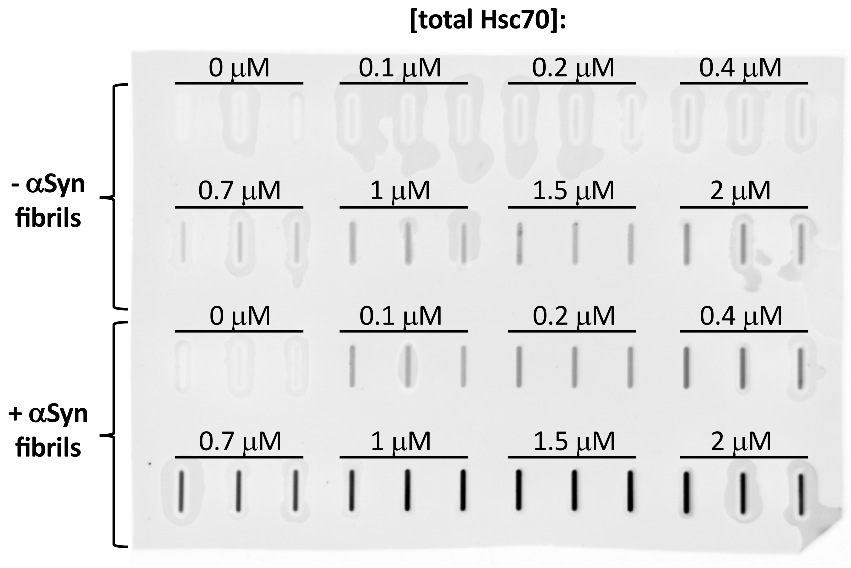

Replicate n°3 (Figure 1B)

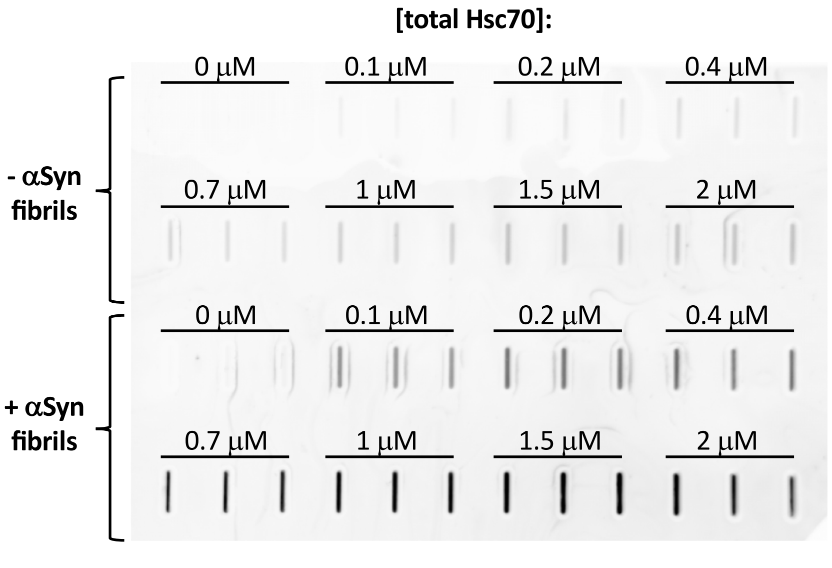

Filter-trap membranes for Figure S5 A-C:

"Quantification of SBD-ATTO488 (A), SBDβ-ATTO488 (B) and SBD-lid-ATTO488 (C) binding to αSyn fibrils"

ATTO488-labelled Hsc70 SBD domain and sub-domains were diluted with the corresponding unlabelled proteins (at a molar ratio 1:50) to different final concentrations (0-5 μM) and incubated with or without αSyn fibrils (1 μM) for 1h at RT. Each sample was then filtered in triplicate through a cellulose acetate membrane and the amount of ATTO488-labelled Hsc70 domain trapped onto the membrane was quantified.

Two to 3 independent experiments were performed, and the corresponding filter-trap membranes are shown. For each domain or sub-domain the replicate presented in Figure S5 is indicated.

SBD, Replicate n°1

[total SBD]:

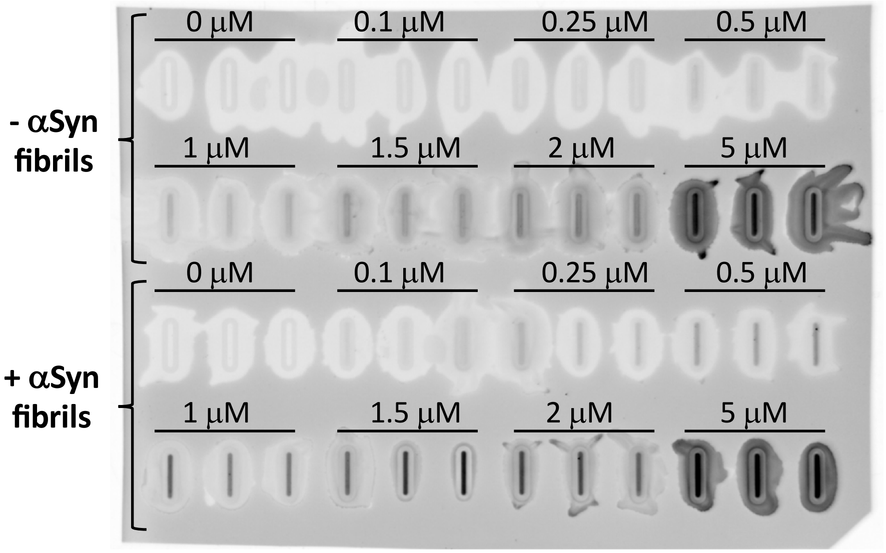

SBD, Replicate n°2

[total SBD]:

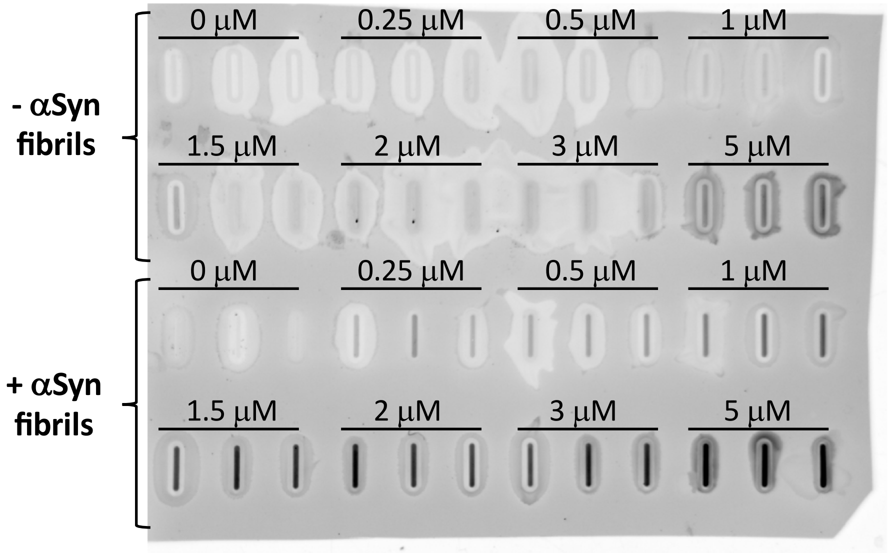

SBD, Replicate n°3 (Figure S5A)

[total SBD]:

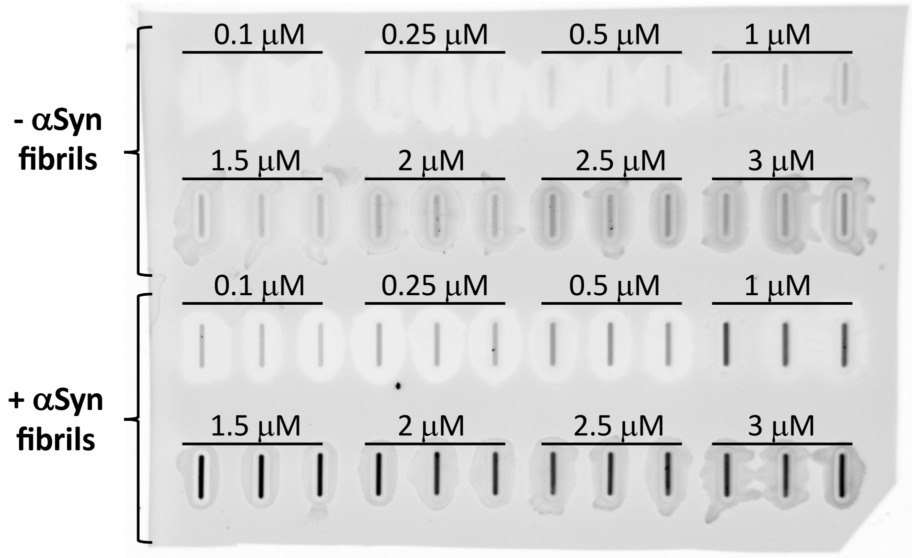

SBDβ, Replicate n°1 (Figure S5B)

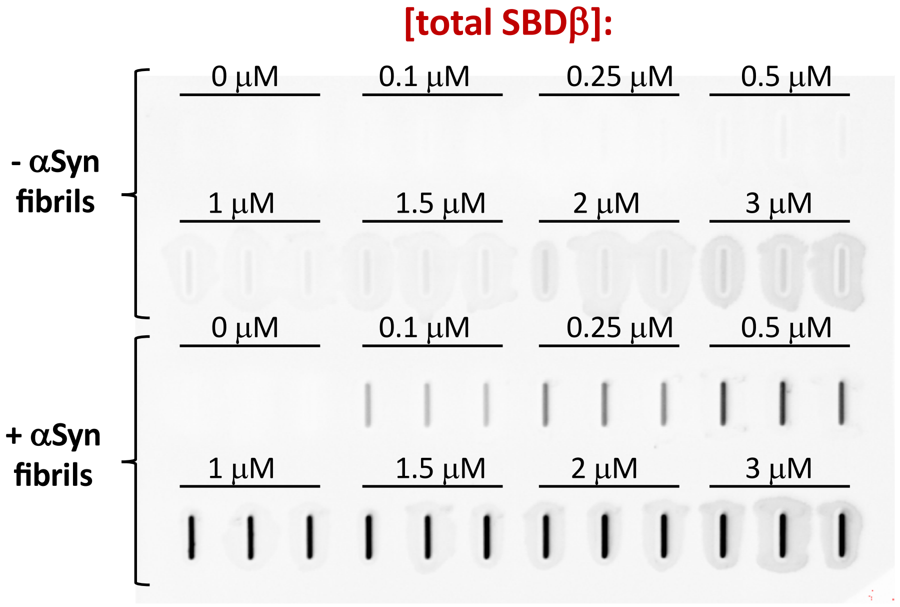

SBD-lid, Replicate n°1 (Figure S5C)

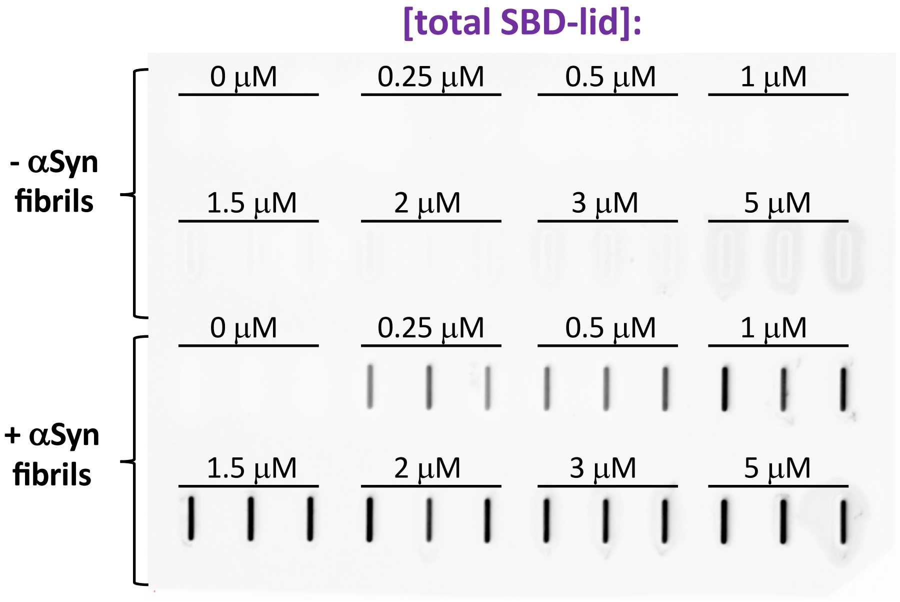

SBDβ, Replicate n°2

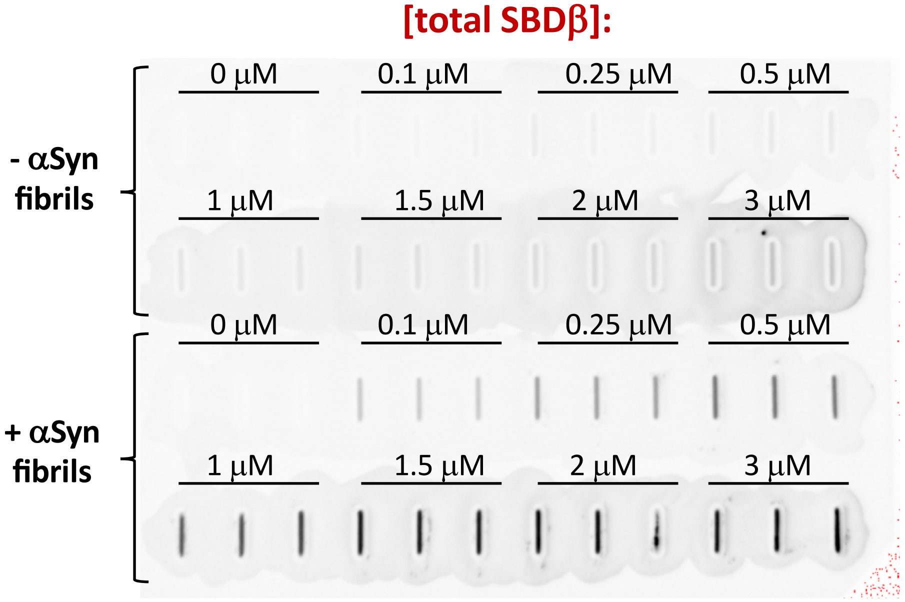

SBD-lid, Replicate n°2

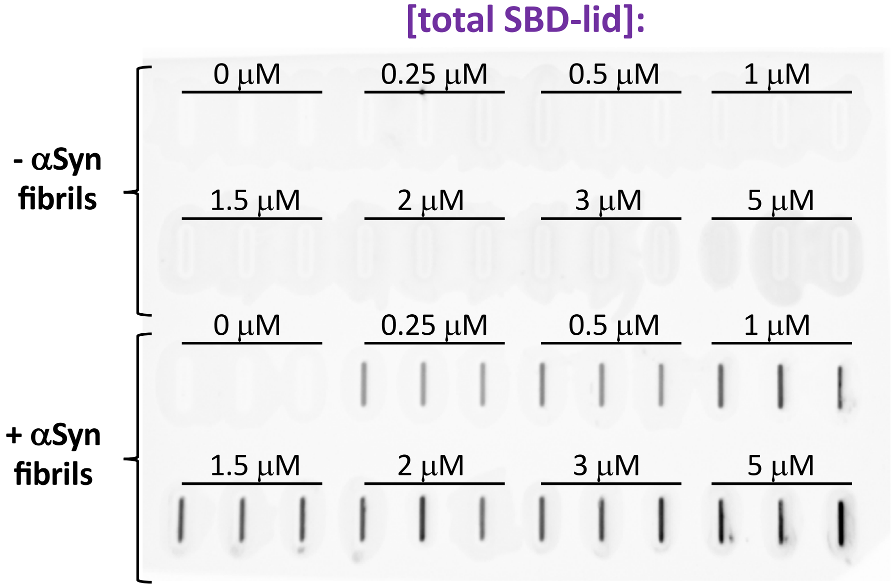

Supplement: S1 Raw images — (PDF) [file pone.0237328.s007.pdf]
