# Supplementary material for: Polypeptides derived from α-Synuclein binding partners to prevent α-Synuclein fibrils interaction with and take-up by cells
Source: PLoS One. 2020 Aug 13;15(8):e0237328. doi: 10.1371/journal.pone.0237328 (PMC7425896; doi:10.1371/journal.pone.0237328)
Supplement: Attachment — Submitted filename: comments-syn-3.docx [file pone.0237328.s008.docx]

**Comments on manuscript titled ”Polypeptides derived from α-Synuclein fibrils interaction with and take-up by cells”**

The study by Monsellier E et. al. is based upon their earlier findings on interaction of Hsc70 and sodium/potassium pump NaK-ATPase with α-syn. In the present study, authors designed various peptides based upon the interaction motif of Hsc70 or NKA, and investigated their potential to bind to a-syn monomer/fibrils, and ability to inhibit fibrils to bind or internalize Neuro-2a cells. They found that two of the Hsc70 based peptides though affected α-syn assembly however could not inhibit its internalization into cells. NKA based peptide did not bind to fibrils however affected α-syn fibril binding to cells without affecting their uptake. Though its an interesting attempt to design small peptides that could affect α-syn fibril uptake, the study primarily could not produce such inhibitors, and thus its not clear about the significance of the study. In addition, I do have following major concerns that must be addressed.

(1) The kinetics of α-syn fibrillation at the α-syn concentration used for interaction assay in Figure 1A is not shown. Also its important to mention that the fibrils used for interaction study were obtained from which phase of fibril formation.

(2) Figure 1B: The protocol followed to measure binding affinity is not clear. The text (Page 5, 1^st^ paragraph) mentions that only labelled Hsc70 was incubated however the figure legend and material and Methods mention that unlabeled Hsc 70 was also added with ratio of 1:50 (lablled:unlabeled)

(3) Figure 1B: The data on filter membrane is shown for 8 different concentrations of Hsc70 however the fitted curve shows 9 data points. Also “Y’ axis is labelled as “% maximal binding” which should be defined in the Figure Legend.

(4) Figure legends for Supplementary Figures are missing at least in the version I received for review.

(5) Figure S1A and S1B: Control showing cells in the absence of α-synuclein fibrils is missing. Also instead of “αsyn” author should mention “αsyn fibrils”. Also control showing the effect of incubation of monomeric α-synuclein with neuronal cells is missing.

(6) Figure 2D and 2E: Reference indicating chloramazine and EIPA as inhibitor of clathrin-mediated endocytosis in Neuro-2a cells should be cited in the text.

(7) Figure 3A: Various peptides, based upon the interacting region of Hsc70 to α-syn, were synthesized. It seems that some of the interacting sites, such as region 531-535 was not explored. Authors should provide a justification for selecting only few of the sites shown in Figure 3A. Similarly, what is the rationale of selecting Hsc-3 peptide.

(8) Figure S5: The chromatogram for peptide alone should also be shown as a control study.
